# The Removal of Westminster Hospital

**Published:** 1912-11-30

**Authors:** 


### THE REMOVAL OF WESTMINSTER HOSPITAL.

The projected removal of the Westminster Hospital from its present site forms the topic of the moment in hospital circles of the Metropolis. The general policy of decentralisation and migration from the no longer residential districts in which several of our London hospitals have been left by changes of time and circumstance has always been strongly advocated in The Hospital; and the adoption of this policy by the authorities at Westminster commands our enthusiastic sympathy and support. Not so very many years ago Westminster Hospital was within a stone's throw of some of the foulest slums in London; and in spite of its situation close to the Abbey, the Houses of Parliament, St. James's Park, and the Government Offices, it yet ministered to a very large and indigent population almost at its very doors. The Horseferry Road and its tributaries are not yet, it is true, entirely cleared of tenement property; but recent years have witnessed an enormous change in the population of the vicinity, and at the present day no one would dream of planting a hospital at Westminster, supposing none had ever existed there. Then, again, there are three large general hospitals within a mile or so of the Westminster: St. Thomas's, St. George's, and Charing Cross. So that the district will still be quite adequately served even after the Westminster Hospital has moved.

It is understood that a Bill is to be promoted in Parliament to enable the present building and site to be sold, and to empower the Governors to buy or lease another site and to re-erect the hospital thereon; and also to deal similarly with the Medical School, the Nurses' Home, and the Incurables Fund. No announcement has been made as to the locality which the Governors have in view; and, indeed, no final decision has yet been taken. It may be surmised that a proposal of such magnitude would not have been made public unless the plans had included some consideration of this important point; but even when the future site is definitely selected the Governors will be wise to keep it a secret until all business negotiations in connection with the lease or purchase are concluded. It will, therefore, probably be some months before any authentic information on this point is available.

The removal of King's College Hospital has so far progressed towards successful accomplishment that it is permissible to wish the authorities at Westminster an equal success in their bold but well-conceived emulation; and we cordially wish them a warm response from the public when the time comes for an appeal for funds, and a benefactor as generous as the Hon. W. F. D. Smith has proved himself to King's College Hospital. There should be no difficulty in realising a handsome sum from the sale of the present site and buildings which face the Abbey. When St. George's, which also occupies one of the finest sites in the West End of London, had a move under consideration several years ago, the main obstacle was the fact that only a portion of the ground was freehold, and that thus a sale would have benefited the ground landlord more than the hospital funds. In the case of the Westminster this difficulty, we believe, does not arise; and we trust there will be keen competition to possess this admirable plot. From the point of view of London it should not be built on at all.

